# Fluorescent nanoplastics: What steps are needed towards a representative toolkit?

**DOI:** 10.1186/s43591-025-00159-0

**Published:** 2025-12-15

**Authors:** Karen van den Akker, Laurens D. B. Mandemaker, Joren M. Dorresteijn, Linda A. Amaral-Zettler, Bert M. Weckhuysen, Florian Meirer

**Affiliations:** 1 Inorganic Chemistry and Catalysis Group, Institute of Sustainable and Circular Chemistry, Utrecht University, Universiteitsweg 99, Utrecht, 3584 CG The Netherlands; 2 Division of Toxicology, Institute for Risk Assessment Sciences, Utrecht University, Yalelaan, 104-106, Utrecht, 3584 CM The Netherlands; 3 Department of Marine Microbiology and Biogeochemistry, Royal Netherlands Institute for Sea Research (NIOZ), PO Box 59, Den Burg (Texel), 1790 AB The Netherlands; 4 Department of Freshwater and Marine Ecology, Institute for Biodiversity and Ecosystem Dynamics, University of Amsterdam, P.O. Box 94240, Amsterdam, 1090 GE The Netherlands

## Abstract

Degradation of plastic waste in the environment induces the formation of plastic particles, that can be either microplastics (MPs, < 5 mm) or nanoplastics (NPs, < 1000 nm). Their presence poses an emerging concern for environmental and human health, but the scale of the risk remains unknown due to the various challenges in their proper detection and identification. Fluorescence-based analytical methods are commonly employed for toxicological and/or exposure model studies, as well as environmental studies. Aiming at researchers assessing the effect and behaviour of NPs within exposure and imaging studies, this review critically explores different strategies for using or synthesizing fluorescent NPs, starting with highlighting relevant overlap from fluorescent MP work, to identifying current knowledge and methodological gaps. Unfortunately, the prevailing strategies for obtaining fluorescent NPs, especially using commercially available polystyrene (PS) beads and dye loading synthesis routes, are inadequate and not representative of environmental NPs, although in recent years promising alternatives have been provided. For that reason, we recommend various approaches for making fluorescent model NP particles. The article ends with concluding remarks and an outlook on the challenges in NP detection, with a suggested “roadmap” to aid the reader in determining the ideal approach of making or using fluorescent NPs in their own field of application.

## Introduction

 Due to low cost, versatility, chemical resistance, and other beneficial properties, plastics are widely used across the globe. In 2019, plastic global production was ~ 368 Mt, showing a great increase compared to the 2 Mt in 1950 [1]. The increasing large-scale production comes at a great cost, because mismanaged plastic waste ends up in the environment. Annually, approximately eight Mt of the plastic enters the ocean, where the weathering conditions cause the degradation of plastic waste into ecotoxic microplastics (MPs) and nanoplastics (NPs) [2]. Still, their abundance and behavior in marine environments are not well characterized [3]. This is primarily due to the wide range of location-dependent plastic concentrations, as well as challenges in MP and NP detection, including proper sampling and separation [3, 4]. 

MPs are defined as plastic particles with a size smaller than 5 mm [4, 5]. For NPs there is still some debate regarding the size class definition. Several researchers define a range of 1–100 nm, following the IUPAC definition of “nanoparticles”, whereas others apply a < 1 μm criterium [5]. In this review article, we adopt the latter definition of plastic particles smaller than 1 μm for NPs, also agreed upon within the European research cluster to understand the health impacts of micro- and nanoplastics (or, the CUSP cluster) [6]. Another important distinction is between primary and secondary micro- and nanoplastics (MNPs) [5, 7]. Primary MNPs are specifically synthesized within these particle size regions for a particular application, such as cosmetics or as model particles for exposure/toxicity assessments [8]. In contrast, secondary MNPs originate from plastic fragmentation events in the environment and from daily use and applications [4, 8]. These secondary MNPs might have a more complex size, shape, and surface group reactivity than primary MNPs and hence are considered a greater threat [9, 10]. Due to the limitations in material availability, most current detection or toxicology studies still focus on primary MNPs, even though research initiatives have explored ways to make model MNPs from degradation processes such as cryo-milling *(vide infra)*.

With the prevailing analytical techniques, it is now possible to detect MPs in the environment or from any sample matrix and to assess (harmful toxic) effects on living organisms [11–16]. Since the relative toxicity of MPs is generally higher the smaller the particle size, one could hypothesize that NPs could be even more toxic [10, 17]. This could induce even greater risks as they are able to diffuse through several biological barriers [18, 19]. The higher surface-to-volume ratio of NPs compared to MPs may allow higher sorption of other toxic pollutants [20]. However, a large methodological gap exists for environmental NP analysis because most optical methods have a size limitation of a couple of micrometers, that is related to the diffraction limit of light [8, 21]. Therefore, it is of utmost importance to find analytical methods that allow for the proper detection of NPs, and great efforts have been made in recent years to detect and identify NPs. To achieve this goal, it is vital to apply complementary analytical techniques, as both imaging the particles and (chemically) identifying them is essential [21]. 

Several analytical techniques are currently available that provide the similar spatial resolution as micro(-spectro)scopic techniques, including atomic force microscopy (AFM), electron microscopy (EM), or photoinduced force microscopy (PiFM) [21–23]. Nevertheless, spatial resolution is not the only challenge, as the detection limits in terms of particle numbers due to low concentrations make it difficult to get statistically robust output. Challenging sample matrices that contain or consist mainly of hydrocarbons also present analytical challenges [21]. Sample preparation through the use of more advanced filtration systems or digestion protocols can help overcome sensitivity limits [24–26]. For an overview of the possibilities and challenges in detecting and characterizing NPs, the reader is referred to reviews by Mandemaker and Meirer, as well as Schwaferts et al. [8, 21].

**Fig. 1:**
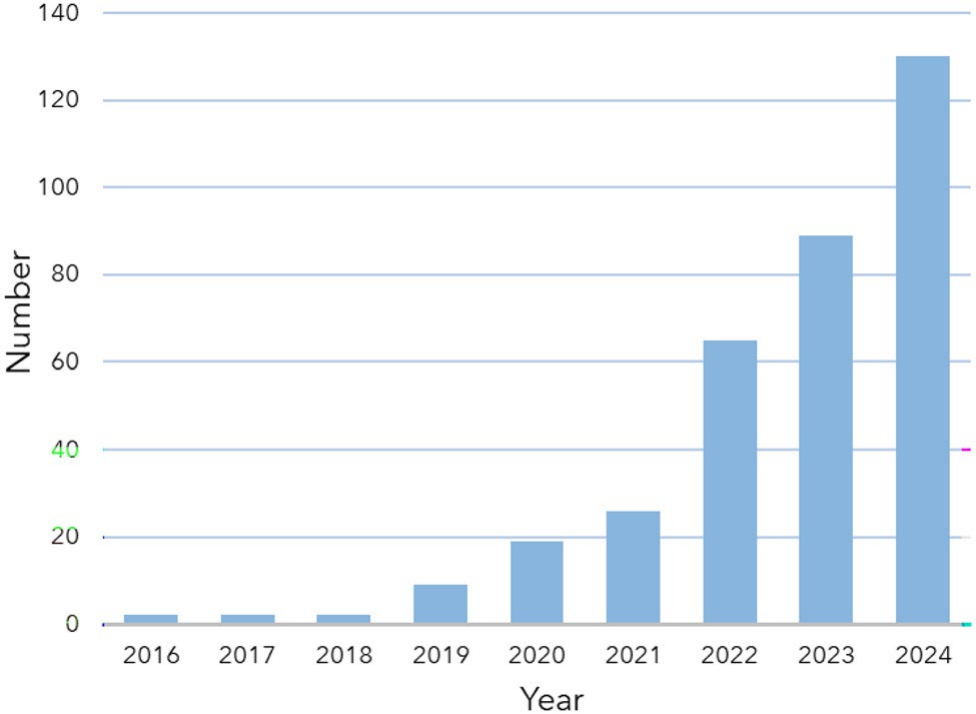
Number of publications on fluorescent nanoplastics (NPs) published per year up to 2025 in the Scopus database, containing *(fluorescent OR fluorescence) AND (nanoplastic OR nanoplastics)* in their title or abstract; all manually verified to be about fluorescent NPs and excluding review articles, perspective articles, or articles that e.g. mention or use (non-fluorescent) NPs but use fluorescence for other purposes

Fluorescence-based analytical methods have great potential for studying spatially complex systems at very high spatial resolution, even enabling tracking of single molecules or (other) nanoparticles. Examples are found in e.g. tracking the diffusion of single molecules through nanoporous catalysts [27] and more specifically, in fluid catalytic cracking catalysts [28], zeolites [29, 30], measuring carbon dots in different environments [31], in live cells [32] or even in protein folding [33]. For MNP related research, primary MNPs can be studied if proper labels are included within the particles, and such studies often focus on uptake experiments of cells or organisms and toxicity assays. Secondary MNPs can be detected if they are stained using fluorescent dyes, ideally with plastic-specific fluorescence, which enables the determination of particle size, shape, type, and count from organic matrices [34–36]. In the past few years, the use of fluorescent NPs has increased exponentially, as illustrated in Fig. 1.

Figure 1 presents the number of papers that were published up to 2025 based on a search in the Scopus database containing (“Fluorescent” OR “Fluorescence”) AND (“Nanoplastic” OR “Nanoplastics”) in their title or abstract. We manually verified that all published work was about fluorescent NPs: e.g. reports on fluorescent detection techniques or toxicological assays that use fluorescent imaging, but do not include fluorescent particles, were not included in this literature study. Additionally, reviews and perspectives were not included in this assay. We excluded studies solely on MPs. These criteria result in a total of 344 publications by the end of 2024 (out of the 485 publications found by Scopus), which is only 7.5% of the 4615 papers in the Scopus database found with the search term “Nanoplastic OR Nanoplastics” in the same time period.

The main drawback of fluorescence-based analytical methods is the required use of fluorescent NPs, that is (usually) not an inherent property of the plastic particles. However, fluorescently labeled NPs help to determine their position and concentration within organisms after exposure in toxicology studies. Moreover, in environmental samples, lipophilic stains, such as Nile Red (NR) can selectively detect MPs [37]. It is expected that the potential of this method extends to NPs, as laboratory studies have also shown successful staining of NPs with NR [38]. In this review article, we explore the following questions: (a) Which fluorescence-centered analytical methods and model particles are currently used for studies on plastic nanoparticles?; and (b) Which fluorescence-centered methods or approaches could be applied in future studies? First, we outline some reported detection techniques involving fluorescent properties and compare their potential and limitations. After that, we briefly summarize examples found in MP detection, focusing on analytical or particle preparation methods that we feel would be suitable to extend to NP focused strategies. Then, we present an overview of the different fluorescent NPs found or synthesized for the assessed literature, addressing the following questions. What synthesis or functionalization techniques were applied? What are the advantages or disadvantages of commercial fluorescent NPs versus the different laboratory-based synthesized NPs? Finally, we present recommendations on which strategies would be most suitable for specific research purposes, including NP toxicity and environmental NP detection studies.

## Detecting fluorescent nanoparticles

A crucial step in acquiring knowledge on NPs, is accurately and precisely detecting them. In this section, a selection of analytical techniques will be showcased that allow the detection of fluorescent NPs. For a broader understanding of these analytical techniques, or to obtain further insights in the advances in pushing the field of fluorescence microscopy towards single molecule observation or achieving Ångström-resolution, the reader is referred to comprehensive works from Combs, Maris et al., Shashkova and Leake, and Reinhardt et al. [27, 32, 39, 40]

### Currently used fluorescence microscopy techniques

**Fig. 2:**
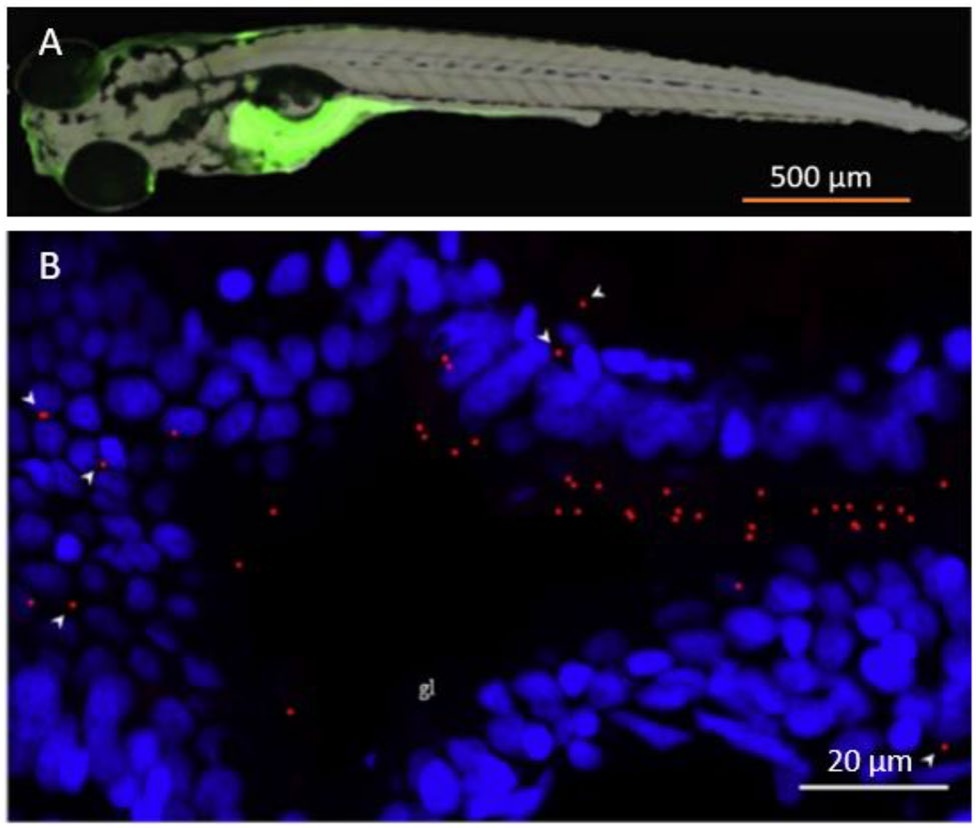
Comparison of stereo-fluorescence microscopy and confocal laser scanning microscopy (CLSM). (**A**) The accumulation of fluorescent 200 nm polystyrene (PS) beads (green) in zebrafish is apparent from the stereo-microscope image. (B) The uptake of 500 nm PS beads (red) in zebrafish with a CLSM, where the cell nuclei have been marked in blue. Images **A** and **B** are adapted with permission from Pedersen et al. and Parenti et al., respectively [46, 47].

Different types of fluorescence microscopy are available, that differ in their working principle and related spatial resolution. For example, stereo-microscopy results in lower resolution and magnification, that makes it unsuitable for single-particle NP detection. Others, like confocal laser scanning microscopy (CLSM) result in much higher resolution and greater contrast [41]. The difference in resolution between stereo and CLSM is demonstrated in Fig. 2. Both images show fluorescent polystyrene (PS) beads in zebrafish with bead sizes of (A) 200 nm and (B) 500 nm in diameter imaged using stereo-microscopy and CLSM, respectively. The stereo-microscope shows where the beads occur in the whole zebrafish, as indicated by the green region. The CLSM allows distinguishing the individual beads in a selected part of the zebrafish. Depending on the research question, both techniques provide valuable information on the uptake and distribution of model plastic particles within organisms (and are in that sense complementary). The stereo-microscope is relatively more available, and easier and quicker to operate, whereas the CLSM provides higher contrast and resolution, but is more advanced to operate and use. Additionally, it should be noted that CLSM still has a lower size limit of 200–300 nm, which might not be sufficient for smaller NPs [42–45]. 

### Promising alternative fluorescence microscopy techniques

**Fig. 3:**
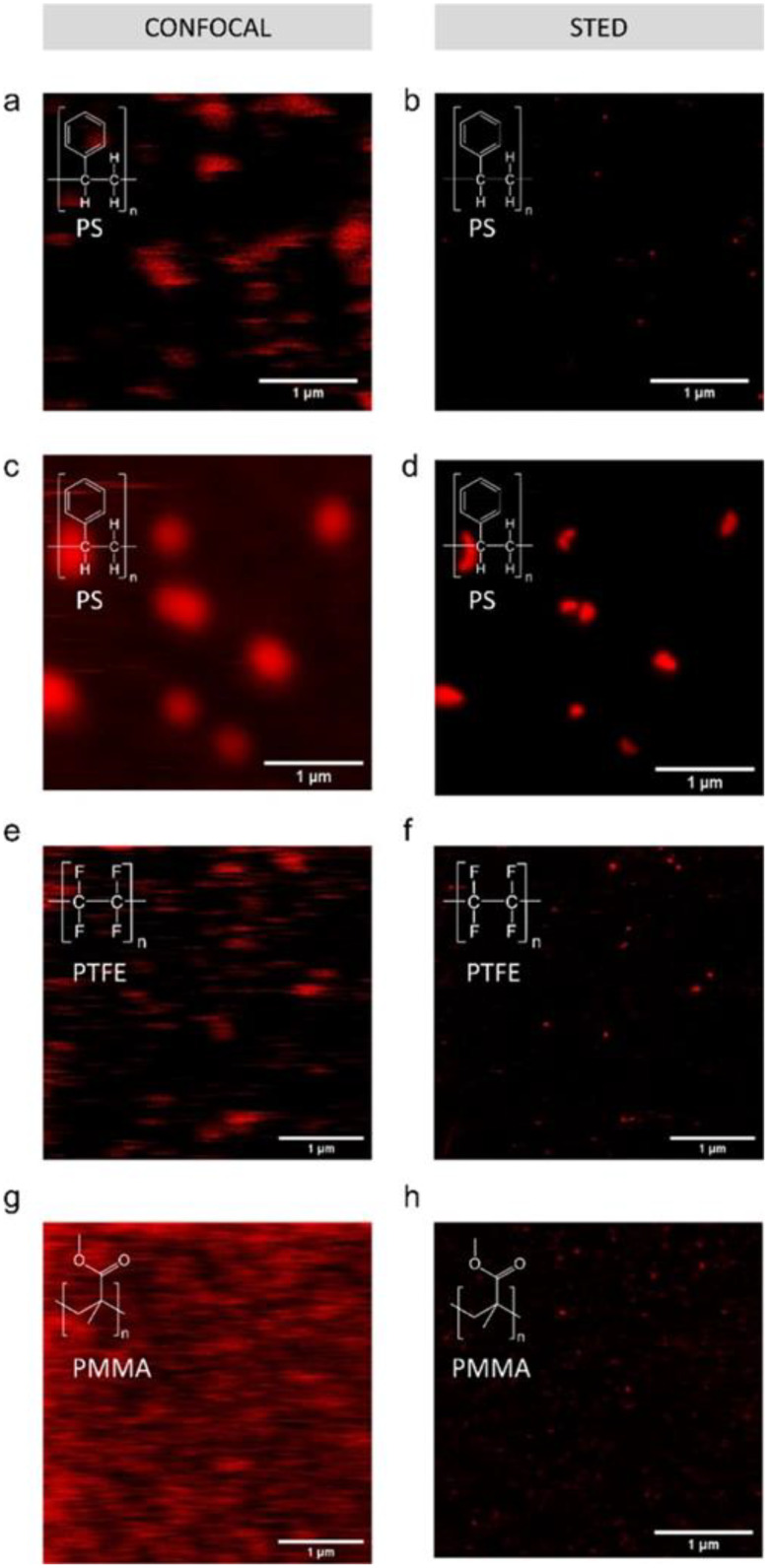
Comparison of **A**) confocal laser scanning microscopy (CLSM) and **B**) stimulated emission depletion (STED) microscopy of different NPs labelled with Atto647N. The polystyrene (PS) particles are secondary nanoparticles (NPs) obtained from a-b) an expanded PS plate exposed to hot water, and c-d) a sanded petridish. The others were primary particles of e-f) polytetrafluoroethylene (PTFE) and g-h) polymethylmethacrylate (PMMA). The same field of view is measured for both CLSM and STED. Reproduced with permission from Nguyen and Tufenkji [49].

A promising fluorescence microscopy technique is stimulated emission depletion (STED) microscopy, as it is capable of surpassing the diffraction limit. For a detailed fundamental understanding of this technique, the reader is referred to a recent review by Vicidomini et al. [48] Harke et al. showed how the resolution of this method can be manipulated, enabling the detection of fluorescent PS beads of 24 nm with much higher accuracy than confocal microscopy [43]. In Fig. 3 the practical difference between the CLSM and STED techniques is highlighted for relevant fluorescent NP particles [49]. In this study, several plastics (for PS, even secondary NPs) were labeled with fluorescent dyes suitable for STED, and their presence in a worm was detected with STED, again with much higher accuracy than with CLSM.

Within the assessed literature, STED and other super-resolution microscopy techniques, such as structured illumination microscopy (SIM), reversible saturable optical linear fluorescence transitions (RESOLFT), or single-molecule localization microscopy (SMLM), have not been utilized yet within the field of (environmental) NP detection, that may be attributed to challenges such as the need for fluorescent labeling, limited applicability in complex and scattering environmental media, relatively low throughput, and higher costs compared to more conventional characterization techniques [50, 51]. However, we believe that these techniques provide great potential for NP detection purposes, as they could provide the concentration, location, and size of the particles.

Instead of relying on one analytical technique, a combination of methods might provide more detailed information about the nature and behavior of NPs. One possible strategy is presented by Molenaar et al., who combined sensitive fluorescence video-microscopy with single-particle tracking (SPT) techniques [34]. In this way, the Brownian motion of individual particles could be assessed, providing their hydrodynamic radius.

In other correlative approaches, fluorescence microscopy could be complemented with transmission electron microscopy (TEM), scanning electron microscopy (SEM), AFM, or other (electron or even X-ray) microscopy techniques [21, 52]. This combination of fluorescence microscopy and EM is mostly used in the field of life science or material science, but also for other purposes [52–57]. Although previously the difference in resolution between fluorescence microscopy and electron microscopy presented challenges for correlative imaging, the development of super-resolution fluorescence microscopy techniques has bridged this gap [58]. What is promising about these approaches is that they can unveil both proper localization and chemical information about the particles. However, within the assessed literature, correlative approaches with fluorescence microscopy were not found. On the other hand, correlative approaches of Raman microscopy with SEM were [59–61]. Schmidt et al. were able to detect 200 nm PS beads with concentrations of 2 * 10^− 3^ µg/L in demineralized water, and 20 µg/L in salt water, demonstrating the great potential of correlating imaging techniques for NP detection [59]. 

We expect that the correlation of (super-resolution) fluorescence microscopy techniques with electron microscopy or AFM might be promising as well, as the benefit of having a technique mapping the actual morphology on one hand, like EM or AFM, and overlaying it with the specific fluorescent signal that identifies the plastics on the other, would be very valuable for environmental MNPs that are not otherwise recognizable solely by shape, but still present within a complex matric or media (after digestion). Additionally, we envisage this will be useful in studying the hetero-aggregation behavior of NPs.

### Fluorescence spectroscopy

Each fluorophore has its own characteristic emission spectrum, but for some fluorophores, like NR, the characteristic spectrum even shifts to different wavelengths depending on the environment *(vide infra)* [38, 62]. Fluorescence spectroscopy can be applied to measure quantitative concentrations using the specific emission spectra of the dyes present within NPs. However, this is not possible for every fluorophore in every environment. For example, when using NR as a fluorescence probe for PS in the presence of salt, at low PS concentrations, the fluorescent peak becomes too broad to use for quantitative purposes as a result of emission quenching caused by chloride ions [62, 63]. Nevertheless, there are several reports in which specific peak intensities in the absorption and emission spectra are utilized to determine the NP concentration in aqueous solutions [36, 64–66]. 

Attempts have been made to use these spectrofluorometric methods for the detection of NPs in different environments such as biological tissues, as was done with 9-(dicyanovinyl)-julolidine, a molecular rotor probe [67]. This semi**-**quantitative approach allowed for a theoretical detection limit of 65 ng/mL when using PS beads of 50 and 100 nm. Interestingly, Costa et al. showed that the ratio between two peaks might also provide quantitative information in different environments [62]. When they used a pyrene probe for the detection of PS on salt, sand, or silica with PS, the ratio between two of the peaks in the emission spectrum provided the PS concentration, with a detection limit of 0.2 µg/g. The main downside of using spectroscopy is the lack of spatial information within e.g. organisms, but this would be less relevant if the sampling method and sample preparation isolates specific matrices or points of interest (e.g., blood, or after digestion of specific tissue). All in all, fluorescence spectroscopy is a straight-forward technique that can be implemented for quantitative purposes if the relevant matrix allows. However, published fluorescence-based assays often report limits of detection (LODs) in the ~ 10–1000 µg L⁻¹ range, that is comparable to or higher than environmentally observed NP levels that frequently lie at sub-µg L⁻¹ to low-µg L⁻¹ in surface and drinking waters. This shows that preconcentration and very proper matrix cleanup will be essential as only a few recent probe systems approach sub-µg L⁻¹ LODs at this stage [67–71]. 

### Flow cytometry

Flow cytometry is widely used in biology for cell population analysis. In exposure studies, flow cytometry can be used to assess the uptake of fluorescent NPs in cells [72–74]. With this technique, an abundance of cells can be studied in an efficient way, allowing for the determination of the percentage of cells that show NP uptake. In addition, the uptake per cell can be studied semi-quantitatively via this approach. Cortès et al. showed that both the percentage and uptake increase with increasing exposure concentration [72]. Further, Kaile et al. studied the use of flow cytometry in analyzing environmental samples for MNPs. By staining the plastics with NR in an appropriate solvent, they analyzed plastics with sizes from 2 μm to as low as 200 nm. Notably, they compared the concentrations found by both flow cytometry and fluorescence microscopy and found significant differences between the two [75]. This highlights the importance of cross-referencing analysis techniques in general, as every method has its own limitations. It should also be realized that flow cytometry merely provides concentrations and no information on particle size and shape; however, this could be done using a flow sorter. In the recent work from Salvia et al., a rapid nanocytometry/flow-cytometry workflow using NR staining to detect and quantify (environmental) NPs in human peripheral blood was reported, showing low backgrounds and measurable NR-positive events across healthy donors, newborns, various patient groups, and mice [76]. Importantly, a core constraint of NR-based assays is that NR readily aggregates/precipitates in aqueous media, creating fluorescent particulates that overlap with true stained plastics and can inflate counts unless countered (e.g., using nanomolar NR, adding DMSO, and gating on yellow fluorescence to separate aggregates from NR-stained particles).

### Recommendations – fluorescent detection

1. Use confocal laser scanning microscopy (CLSM) for (exposure and) toxicology.
2. Characterize fluorescent NPs (quantitatively) with spectroscopy.
3. Explore promising super-resolution techniques, such as stimulated emission depletion microscopy (STED), for NP detection.
4. Utilize correlative techniques to obtain more and decisive information.

### Fluorescent microplastics

Compared to NPs, MPs have received a greater amount of consideration so far, as indicated by 1498 publications in the Scopus database found using Microplastic(s) AND Fluorescent OR Fluorescence up to and including 2023, versus 485 for Nanoplastic(s) AND Fluorescent OR Fluorescence (disregarding the manual selection and verification applied as used and explained for Figs. 1 and 10). To develop better fluorescence-based methods for NPs, it is, therefore, logical to build upon MP research where possible. For that reason, we discuss relevant strategies in which fluorescent properties were exploited in this section.

### Overview of staining approaches

Staining approaches provide an attractive strategy for MP detection in environmental samples. Because the particle sizes of MPs surpass diffraction limitations, the spatial resolution is sufficient to retrieve information on the particle morphology. Often, these staining and analysis rates are fast, allowing high-throughput quantification if coupled with semi-automized quantification software [37, 77]. Hence, this makes the method suitable for larger-scale implementation to detect secondary MPs. However, it is a challenge to find the appropriate dye, since the surface tension and properties are different for each kind of plastic. For that reason, most dyes are only suitable for a range of polymers [77]. Additionally, in efforts to increase the reproducibility, it is essential to prevent co-staining of non-plastic inorganic and organic particles such as shells or wood in water samples [78–80]. That is why one major focus of studies on MP detection is discovering which dyes selectively stain which plastics.

To illustrate, Maes et al. briefly compared the adsorption on plastic and fluorescence intensity of NR, Oil red EGN, Rose Bengal, Eosin B, and Hostasol yellow [81]. Based on these criteria, NR was selected for the development of the staining approach in environmental samples. One study that investigated multiple dyes more extensively was done by Prata et al. [80]. They identified the fluorescence of 8 different lipophilic staining dyes, on dry PS, low- and high-density polyethylene (LDPE and HDPE, respectively), polyvinylchloride (PVC), expanded polystyrene (EPS), polypropylene (PP), polyethylene terephthalate (PET), nylon, and cellulose acetate (CA), in addition to textile fibers. The dyes were tested at two wavelengths, namely 254 and 365 nm. NR was found to be successful in staining most plastics, as from the current selection, only PVC and PS did not result in high fluorescence. Additionally, several dyes were found to selectively stain some plastics. For instance, the dye Safranin-T selectively detected CA, while the dye Acridine Orange selectively detected both PET and CA.

**Fig. 4:**
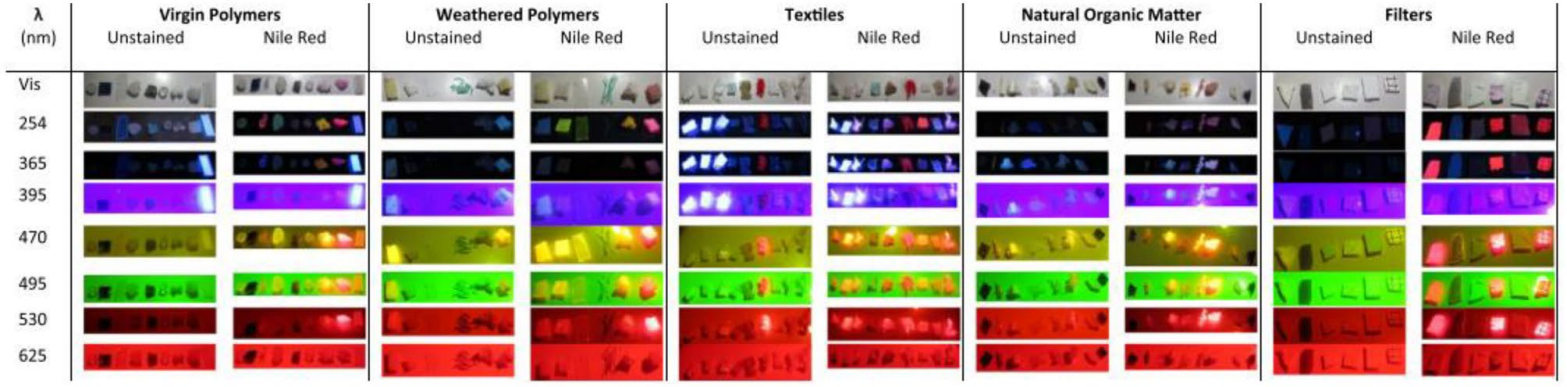
Fluorescence of different nile red (NR)-stained materials under a range of wavelengths. From left to right: Virgin polymers: low density polyethylene (LDPE), high density polyethylene (HDPE), polyethylene terephtalate (PET), polypropylene (PP), polyvinylchloride (PVC), polystyrene (PS), expanded polystyrene (EPS), cellulose acetate (CA), nylon. Weathered polymers: PE, HDPE, PP, PE fibers, EPS, CA. Textiles: cotton, polyester + cotton, polyester, viscose, nylon, wool, rayon, linen, polyamide. Natural organic matter: algae, driftwood, feathers, shrimp shell, shrimp muscle, fish muscle, shell, carbon. Filters: C18, black polycarbonate, glass fiber, mixed cellulose esters, quartz, nitrocellulose. Adapted with permission from Prata et al. [80]

Figure 4 shows the results of dyeing all tested materials with NR and excitation at different wavelengths. Based on this, they also found that NR is mostly selective towards plastics as it would not stain most (in)organic matter, e.g., algae or shells. However, shrimp and fish muscle did show fluorescence in the presence of NR, which highlights the importance of pre-treatment steps before analysis. Often, strong acid or base treatment is advised in this respect, although it can alter the surface groups of the plastic [25]. This might affect the interaction of the plastic with the fluorophore. One different approach that could be considered is to use an additional DNA stain, such as SYBR green, to differentiate between plastic and organisms [75]. One other major finding by Prata et al. is that exposure to weathering conditions greatly influenced the fluorescence properties, as a result of the changed surface chemistry. This should especially be taken into account when studying NPs, as they have a larger specific surface area and hence weathering would induce more chemical change.

In a recent study, Tong et al. demonstrated the potential of staining PE, PP, PVC, PS, and polyurethane (PUR) with Rhodamine B as the dye molecule [82]. All plastics yielded high fluorescence intensity after dry samples were stained with Rhodamine B dissolved in ethanol. Other solvents, distilled water, and acetone were tested as well, but ethanol was most successful in staining plastic. After being stained, most particles were stable during the 24 h they were exposed to different conditions, such as light, KOH, HNO_3,_ or NaCl. However, for practical implementation it must be realized that using ethanol as a solvent for staining requires a work-up step for environmental samples, as the dried (and preferably separated) particles are preferred. Unfortunately, the samples prepared in water were not studied using fluorescence microscopy.

NR and pyrene were used and compared by Costa et al. in their efforts to detect PS and PS-COOH on sand, silica, and salt surfaces [62]. Where the presence of PS caused a peak shift for NR, the peak position remained the same for pyrene. However, for pyrene, the ratio between the intensity of two peaks/vibronic fluorescence bands changed proportionally to the PS concentration. This allowed sensitive detection of 0.2 µg/g on salt and 2 µg/g on sand. In efforts to extend this approach to secondary plastics, the authors highlighted that the results for pyrene remained similar for PS-COOH, indicating the independence from surface groups [62]. 

Lv et al. performed another comparative analysis between dyes on PE, PS, PVC, and PET [77]. Instead of regular staining, they added a thermal treatment step, which improved their procedure because of thermal expansion and contraction properties. They tested NR (excitation at 460 and 543 nm), Safranine T (excitation at 520 nm), and fluorescein isophosphate (excitation at 488 nm) dyes. They also took great care in selecting the right dye concentration to prevent quenching by dye aggregation. All of the dyes resulted in high fluorescence, even PVC. In the previously discussed study by Prata et al., NR did not stain PVC, underlining the appeal of the thermal approach, for it enabled staining of less hydrophobic plastics. Interestingly, the dependence of fluorescence intensity on temperature varied per plastic. Therefore, combining different procedures might also enable discriminating between plastics.

Karakolis et al. also employed a heating step to stain a great range of plastic with low cost dyes from the textile industry [83]. This approach was suitable for PS, LDPE, HDPE, PET, PVC, PP, and polyacrylonitrile (PAN). However, of the three dye molecules they used, two were toxic for *Artemia salina* as they increased the mortality rate significantly after exposure, making them unsuitable for toxicology studies. This is unfortunate, as the dyes behaved differently and would have allowed for investigating different polymer types within one sample. However, the nontoxic iDye pink showed high fluorescence intensity compared to NR for all plastic types except PP and LDPE, but even for these types, it was still usable. The fluorescent particles remained stable under saline conditions, during KOH treatment, photobleaching, and exposure to oil, as heating above the polymer glass transition temperature traps the dye in the polymer matrix. It should be noted that dry particles were used, so it is unclear if this approach would be suitable for environmental samples. Additionally, the selectivity of the method is unresolved, although it was found that the dyes did not show fluorescence when they were just dissolved in water [83]. However, for risk assessment studies, this could be an interesting approach, if it works for NPs as well and the toxicity is not an issue.

Overall, dye-based staining coupled to fluorescence imaging offers a practical, high-throughput route for screening and quantifying MPs, but its reliability depends on matrix-specific pretreatment and careful dye selection to avoid co-staining, aggregation/quenching, and polymer-dependent variability introduced by weathering and thermal exposure. Emerging strategies, such as thermal-assisted staining, multi-dye panels, and orthogonal counterstains for biogenic material, are promising, yet require standardized QA/QC and confirmatory polymer typing (e.g. using µ-Raman/FTIR micro-spectroscopy) to ensure specificity across diverse samples. These considerations will become even more critical when extending protocols to NPs, where higher surface area and stronger matrix interferences amplify both false positives and characterization limits.

### Nile red staining and its versatility

Generally, Nile red (NR) is considered to be the most suitable staining dye due to its solvatochromic nature and high fluorescence intensity [75, 81, 83]. Shim et al. were the first to identify and quantify MPs with NR staining in 2016 [78]. This was followed by Maes et al., who established and validated a screening method with NR in 2017 [81]. To date, NR staining has been applied multiple times to detect MPs in environmental samples [37, 84, 85]. For instance, Erni-Cassola et al. successfully utilized NR to detect MPs in surface seawater [37], Ferraz et al. employed it to detect them in river water [85], and Ranjan et al. successfully analyzed the release of MPs in disposable coffee cups applying an NR approach [84]. Due to this wide usage, prior research has explored the limits and possibilities of NR-based methods.

For example, Nel et al. demonstrated that to improve the quantitative accuracy of this approach, great care should be taken in selecting the fluorescence threshold [86]. They determined that the fluorescence brightness depended on polymer type, size, color, shape, and staining procedure. When trying to exclude non-polymeric materials, these factors should therefore be taken into consideration for defining the threshold detection limit. Since NPs are even smaller and of lower fluorescence intensity than MPs, we anticipate that this step is critical for NP detection as well.

The staining procedure is another crucial step for MP detection. To achieve high fluorescence, a sufficient amount of NR should be used, but in case of an excess, the resulting aggregation is known to quench fluorescence. Additionally, the solvatochromic nature of NR demands careful consideration of the solvent and temperature. Konde et al. made great efforts to develop an optimized staining procedure, resulting in using a 1:1 mixture of ethanol and acetone with 20 µg/mL NR, to stain the plastics for 10 min at 50 °C [87]. It should be noted that this was determined for PE, PP, PET, and PVC, so other plastics could yield different results.

For a comprehensive fundamental understanding of NR, the reader is referred to a study by Sturm et al. [79]. In this work, the authors tried to increase the selectivity of NR for plastic by using three derivatives, either more hydrophobic or hydrophilic. The results were not as expected, but a mixture of regular NR and NR where the ethyl groups were replaced with branched ethylhexyl groups at pH 2.5 was found to give the best selectivity for MPs. However, they emphasize that pretreatment of environmental samples for the removal of organic matter remains critical [79].

**Fig. 5:**
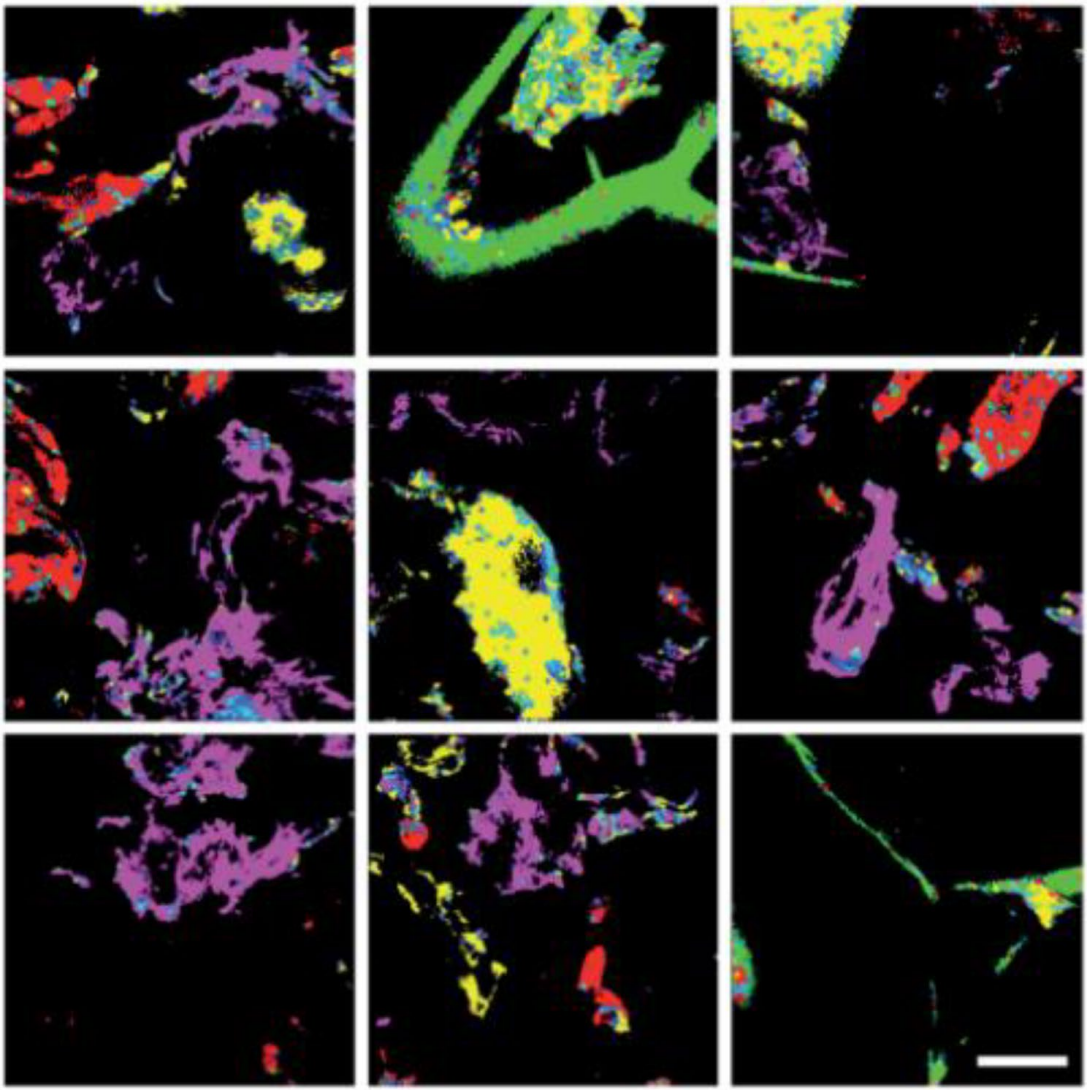
Identification of Nile red (NR)-stained microplastics (MPs) based on fluorescence lifetime analysis and phasor plot. Green: low density polyethylene (LDPE); Yellow: polystyrene (PS); Pink: polyethylene terephthalate (PET); Red: Nylon. Scale bar of 200 μm. Reproduced with permission from Sancataldo et al. [88]

Another attempt to increase the selectivity of NR was done by Sancataldo et al. [88]. They reported a polymer-dependent fluorescent lifetime. This lifetime imaging in combination with phasor analysis allowed for the identification and quantification of plastics (i.e., LDPE, PET, PS, and nylon) within an aqueous sample. Figure 5 shows that different MPs within one sample can be distinguished from each other via this method. One more advantage lies in the fact that this footprint is independent of the NR concentration, which typically provides a great challenge. These investigations, however, fall short of addressing the performance for samples with plastic concentrations representative of environmental conditions, as a rather high concentration of 25 mg/mL was assumed in this study. The effect of particle size on the lifetime should be further investigated, to examine if this method extends to NP detection. It might be interesting to study whether this method allows for distinguishing between non-plastic inorganic or organic matter and plastic materials. If that is a possibility, pre-treatment of an environmental sample might be omitted. This method shows great potential for the detection of environmental plastics and should be considered and further exploited in further research.

In recent work from Nihart et al. focusing on human brain tissue samples, Nile red staining was employed to visualize the morphology of particles found in samples from decedent humans [89]. Especially the supporting information of this work reveals the fluorescent microscopy images, showing micro-sized particles, fibers, and fragments in several samples, after staining with Nile red in a methanol solution. Unfortunately, the fluorescent spectra were not reported, and the authors do not utilize the polymer-specific staining properties of NR. They do use FT-IR microspectroscopy to seemingly measure the stained particles and confirm their polymer identity, which highlights the strength of correlative spectroscopic methods. For future studies, not specifically on this work, it would be interesting to see if the IR spectra recorded on identified particles would also correlate to the fluorescent spectra or lifetime after the Nile red staining, as that would give an ever stronger confirmation of the polymer type found in such delicate samples.

### Extension to nanoparticle staining

Due to their small size, NPs greatly differ from MPs in terms of (Brownian) motion, light phenomena, surface-to-volume ratio, hetero-aggregation, bio-uptake, and release of additives [7]. Mostly as a result of the lower mass and higher surface-to-volume ratio of NPs compared to MPs, not everything that applies to MP staining is automatically valid for NPs. However, we expect that some observations encountered during studies on MPs could be relevant for studies on NPs.

The importance of surface effects highlights the fact that secondary MPs show different fluorescent properties than primary MPs [80]. We anticipate that this is the case for NPs as well. Since staining dyes are most suitable for detecting secondary plastics in environmental samples, the presence of surface groups is a major consideration in selecting an appropriate dye for NPs.

In the assessed literature for this work, only NR has been used for NP detection. To bridge the knowledge gap between MPs and NPs, several studies would be of high interest. The potential of different dyes should be evaluated on (secondary) NPs of different natures, as has been done for MPs. In this search, lifetime imaging methods should also be considered to enhance selectivity. The effects of thermal treatment should also be investigated for NPs, for it greatly enhances fluorescence intensity in MP samples. However, during thermal treatment, the effects on surface groups and NP structure should be inspected. It is expected that the same factors play a role in fluorescence intensity and stability for NPs, but there might be differences in optimal conditions. Importantly, in all of these studies, the aggregation and hetero-aggregation behavior of NPs should be kept in mind.[8] Based on the gained insights, an optimal staining procedure can be determined for NP detection.

### Recommendations - fluorescent staining

1. Study fluorescence lifetime of different materials, including non-plastic organic or/and inorganic matter, to allow chemical distinction between the stained materials.
2. Investigate the effects of thermal treatment on MNP staining, including fluorescence intensity and MNP shape and surface.
3. If needed, optimize the sample preparation using filtration or digestion to remove the majority of the (environmental) matrix.

## Fluorescent nanoplastics

As most plastics do not exhibit autofluorescence, synthesis or treatment steps should be carried out to provide fluorescent NPs as a reference or model material. In this section, we will discuss fabrication methods for fluorescent NPs. There are several ways to obtain fluorescent NPs. Currently, the most common approach is to purchase fluorescent PS spheres, which are likely synthesized by an emulsion polymerization, but other strategies include lipophilic staining, nano- precipitation, or emulsion polymerization in the lab. Table 2 presents the advantages and disadvantages of each of these methods. The following sections discuss these on a deeper level and place them in the context of NP detection.

**Table 2:** Methods for obtaining fluorescent nanoplastics (NPs), including the related advantages and disadvantages in terms of usage or application

Method	Advantages	Disadvantages	Examples found in:
Commercial	Ease of handling Comparative if obtained from the same supplier Reliable Very narrow particle size distributions	Leakage of dye Predominantly PS Primary NPs Hardly any information available regarding chemicals, synthesis procedures etc.	[46, 47, 64, 66, 72, 73, 90–109]
Staining	Simple operation Suitable for secondary NPs All plastic types Operation after exposure	Suitable dye needed Co-staining lipids Sensitive to surface groups	[34, 38, 110, 111]
Nanoprecipitation	Large variety of plastic types, from any source Additional compounds/markers can easily be added	Surfactants often needed for stability Primary NPs Encapsulation of dye might lead to leaching in organic media	[65, 112–114]
Emulsion polymerization	Stable – dye implemented in structure Variety of plastics	Primary NPs Polymerization of several common plastics difficult within emulsion Might contain (toxic) monomers after synthesis	[115–117]

### Commercial fluorescent nanoparticles

**Fig. 6:**
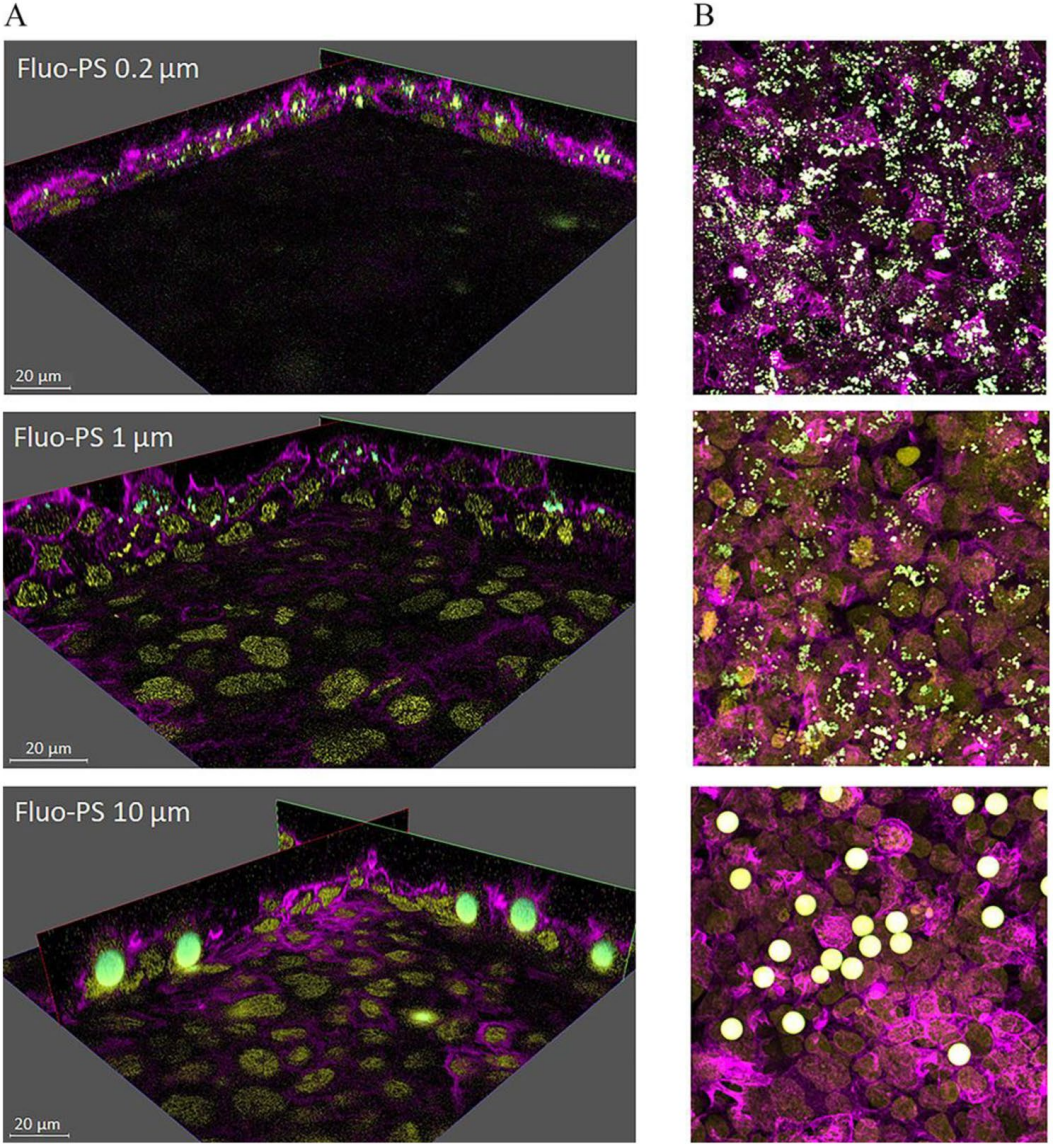
Fluorescent polystyrene (PS) beads (green) of different sizes internalized in placenta cells. Fluorescent stains were used to stain the nucleus (yellow) and β-actin (purple), together with **A**) a 3-D stack of images generating a complete view of the location of the particles within the cells. **B**) Maximum projection images of the respective sample. Reproduced with permission from Dusza et al. [95]

Their ease of handling, avoidance of an extra synthesis procedure, and well-defined properties (such as dispersion and concentration) make commercially available NPs an attractive choice. An elegant example is highlighted in Fig. 6, which illustrates the internalization of fluorescent (weathered and pristine) PS spheres of different sizes in placenta (BeWo b30 choriocarcinoma) cells [95]. In this specific work, fluorescent dyes also stained the cell compartments (the nucleus and β-actin) and 3-D scans visualized the uptake and internalization of the particles within the cell. Although size-dependent uptake and cellular transport were observed, the cell viability remained unchanged for all particles. For these specific examples, commercial model fluorescent NPs can play a pivotal role in understanding the potential health risks of MNPs in early life [118]. 

There are many examples using fluorescent PS spheres to study uptake behavior, and a great variety of sources have been used in different studies, but information regarding the commercial synthesis method is often hard to retrieve. This makes it difficult to understand important potential differences between the beads, such as their stability, which largely depends on the interaction between fluorophores and NPs, and surface chemistry.

To illustrate, Catarino et al. used PS NPs from Degradex Phosphorex, where the fluorescent dye is physically adsorbed on the particle surface [92]. The authors revealed that this dye leaches from the nanoparticles and accumulates in the organism, leading to false indications of the number of nanoparticles based on fluorescence intensity. However, in their conclusion, they reasoned that all fluorescent particles will give the same inconclusive results, without considering the specific interaction between the plastic and fluorophore. Clearly, inherently reversible, physical adsorption is a relatively unstable labeling method, as the interactions are weaker than a chemical bond and highly dependent on the matrix / medium (conditions) in which the particles are dispersed. In this system, it would therefore be likely that favorable interactions between the dye molecules and tissue in the organism resulted in an equilibrium shift and consequent dye leaching.

Some companies do mention their general method for synthesizing the fluorescent NPs. For example, Bangs Laboratories manufactures internally dyed fluorescent PS particles via a solvent swelling/dye entrapment strategy [119]. In this method, previously produced polymer beads are added to an organic solvent, which induces swelling of the plastic nanoparticles. Consequently, a water-insoluble fluorescent dye diffuses into the polymer matrix and is entrapped after removing the solvent, for example by transferring to an aqueous phase or through evaporation, which reinstates the original particle size [111]. With this technique, the surface groups remain available, and the particles are bright because of the high possible loading. The photostability of these particles is another mentioned advantage. However, in light of the synthesis method, the stability of the beads may depend on the environment and differ in organic solvents. Thermo Fisher Scientific also produces internally dyed PS beads. Their dye incorporation method may be based on the same principles as the strategy from Bangs laboratories, but this remains unclear due to the lack of data. However, one of the stated advantages of the Thermo Fisher Scientific beads is the prevention of leakage in aqueous media [120]. Nevertheless, Schür et al. demonstrate that the Thermo Fischer Scientific particles show leakage of fluorescent dye when they are in an ultrapure water solution [97]. They realized that it cannot be generalized for all particles from different vendors and that it depends on the experimental conditions. Ultimately, they advocate for testing the stability of fluorescent NPs under different conditions and recommend using covalently attached fluorophores. This again highlights the importance of knowing the fluorescent labeling method. For other suppliers, the information is harder to retrieve. Spherotech discloses that their fluorescent PS microspheres are produced either by staining of PS NPs with a fluorophore solution or by polymerizing a fluorophore in styrene in the presence of PS core particles [121]. These different methods from the same company would already result in different stabilities and surface properties, and it is unclear how they relate to other brands. The company NanoCS offers fluorescent PS beads labeled with various fluorophores, such as rhodamines [122]. For a significant number of companies, no information is provided regarding the synthesis route.

Next to the lack of information regarding the synthesis methods, another main disadvantage of commercial fluorescent NPs is their limitation to predominantly being PS, followed poly(methyl methacrylate (PMMA). This is not representative of the abundance of different types of plastic in the environment, as will be discussed *(vide infra)*, and it is unclear if e.g., their polymer-specific toxicity might vary [1, 11]. 

### Fluorescent staining

Staining plastic with a fluorescent lipophilic dye is a common technique in plastic detection [34, 38, 62, 77–82, 87, 88]. As discussed before, the solvatochromic dye NR is often used to selectively stain plastic particles in the current selection of literature, as result of its high fluorescence intensity. Due to the solvatochromic and lipophilic properties of NR, it gives a strong fluorescence signal when it is attached to a hydrophobic object, such as plastic. Therefore, NR is able to distinguish between inorganic matter like stones and plastic particles, or even between stained organic matrices [34, 38, 79–81]. Although mostly used for MP detection, some studies use NR staining for NPs as well [34, 38, 110]. 

Gagné et al. used NR staining to detect PS NPs in biological tissue [38]. In this study, *Hydra attenuata* was stained with NR after exposure to PS NPs. As Sancataldo et al. illustrated using different MPs [88], the solvatochromic properties of NR distinguish different organic materials; as the emission wavelength was different for the lipids (located at ~ 660 nm) of the organism and PS NPs (located at ~ 623 nm). The intensity of the NP peak correlated linearly with the NP concentration, but because of a slight overlap with the lipid peak, the authors concluded that this method is mostly semi-quantitative. They also observed a higher peak intensity for the lipid peak after the addition of NPs, indicating that NPs could interfere with lipid determination. It would be of high interest to investigate if this method also works for secondary NPs. Another limitation is the inability to visualize the NP accumulation within the organism.

Wei et al. used a similar NR staining approach to detect 50 nm PS NPs in anaerobic granular sludge for wastewater treatment [110]. They also performed this task after exposure, but instead of analyzing fluorescence intensity at different wavelengths, they mapped the resulting fluorescence with fluorescence microscopy images. To clarify, their main focus was the location and not the concentration of the PS beads. Unfortunately, they did not specify how they discriminated between possible organic matter being stained and the added NPs. This is quite critical since they might both be hydrophobic and sensitive to NR. It is possible that the authors assumed no lipids would be visible at the selected excitation and emission wavelengths of 460 nm and 525 nm, respectively. To validate the procedure, this could be confirmed with spectroscopic analyses [38] or e.g., CLSM with spectral imaging in which full spectra are recorded on the different particles or entities mapped. Note that if the NPs were stained with NR prior to exposure, the non-covalent nature of the interaction could result in diffusion of NR into the lipids as well, although this rate would be influenced by the conditions of the procedure.

Molenaar et al. applied NR staining to visualize primary PS NPs in solution. With their combination of fluorescence microscopy and single-particle tracking, they were able to detect PS NPs at very low concentrations, as discussed before [34]. In addition to confirming that NR staining results in detectable fluorescence intensity, they also highlighted that this intensity is dependent on particle size, as it scales with particle surface area. From their linear correlation between intensity and surface area, they draw the conclusion that dye adsorption occurs on the surface, and accumulation inside the particle is not present. Interestingly, based on this principle they were able to distinguish particles of different sizes in a mixture from their fluorescence intensity. However, as the intensity of NR depends on the chemical nature as well, it might not be suitable for mixtures of different plastics. Theoretically, this problem could be avoided if both the intensity and the full fluorescent spectrum and lifetime are assessed [88]. 

Although there has not been a significant number of studies yet on ‘real-life’ NP detection with NR staining, the method offers high potential due to its ability to discriminate between materials. From the fluorescence-based methods reviewed here, NR seems to be most compatible with (secondary) NP detection in environmental samples. For toxicology studies, the presence of hydrophobic lipids or tissues, which are also sensitive to NR staining, should be considered. Therefore, we recommend examining to what extent these parts of the cell can be discriminated from NPs with the solvatochromic properties of NR when it is chosen as a dye. In parallel, it is vital to also search for other dyes that could (selectively) stain NPs and aid in their analysis, and it is expected that new dyes might be synthesized to fill in this role. A promising example is found in the work from Xing et al., in which a tetraphenylethylene functional group with a fused xanthene as fluorophore was employed to stain PS and show exceptional affinity for the plastic, even better than NR. The stained plastics were measured in bean sprouts and HeLa cells [123]. 

In a different, and perhaps more effective, approach, covalent tagging is used to bind fluorophores more strongly to the plastics, making leaching less likely and further changing the fluorescent properties of the dye leading to potential polymer characterization. Merdy et al. used cyanine-3 phosphoramidite, rhodamine-6G, fluorescein sodium salt, and Vat Red 15 to label PP, LDPE, HDPE, PS, PET, and PVC particles. Fluorescein was the most effective in exhibiting specific fluorescent properties depending on the polymer, showing differences for PP, PVC, HDPE, LDPE, and PS [124]. The earlier highlighted work from Nguyen and Tufenkji that compared STED to CLS in Fig. 3 used N-hydroxysuccinimide (NHS)-terminated Atto 647 N, that covalently binds to the amine group of the model aminated-PS spheres [49]. They report that the dye seemed more stable than Nile red when exposed to mineral oil. It should be noted that to quantify the covalent bond, and the amount of bonding versus potential physisorbed or absorbed dye molecules, is challenging. Additionally, although they are covalently bonded, they are still likely on the surface and therefore also prone to chemical interactions with the media to which they will be exposed.

### Synthesis of fluorescent nanoparticles

In other studies, fluorescent NPs were obtained through a synthesis procedure. For this, three different strategies were applied: dye encapsulation and nanoparticle formation via nanoprecipitation and monomer incorporation via either conventional emulsion polymerization or mini-emulsion polymerization. More techniques, such as emulsification-solvent evaporation, are available, but for more insights in this technique the reader is referred to an elaborate review by Reisch and Klymchencko [125]. Generally, these methods allow variability in the plastic type and size. It is important to note that great care should be taken to choose the right fluorophore and its concentration to obtain optimal fluorescence intensity and stability. In the review by Reisch and Klymencho, these factors are also discussed in great detail. It is important to mention that the three methods described in this review are done so generally, reviewing published work that included fluorescent labelling in their synthesis, and not specific aimed towards the difference per polymer type. However, there are essential polymer specific factors to consider. The nanoprecipitation that will be discussed works for most of the polymer types that can properly be dissolved; the emulsion polymerisation techniques work only for polymers that can be produced in conditions supporting the emulsion. For example, PP is produced through catalytic polymerization, whereas e.g. PET and PUR are produced by polycondensation reactions. Both of these processes are hard to combine with emulsion chemistry, so their reference particles are better formed using nanoprecipitation [126, 127]. For PMMA, PS and even PE (using e.g. nickel-based catalysts) their (catalytic) polymerization can be performed in emulsion, and hence they are more suitable for the emulsion polymerization techniques [128, 129]. Finally, as introduced before, laser ablation and cryo- or ball milling techniques can be used to generate secondary NPs from larger plastic (beads) [130–133], but these then require additional staining steps to obtain fluorescent properties. Another very promising option would be to generate secondary particles from bulk fluorescent plastics.

#### Nanoprecipitation

**Fig. 7:**
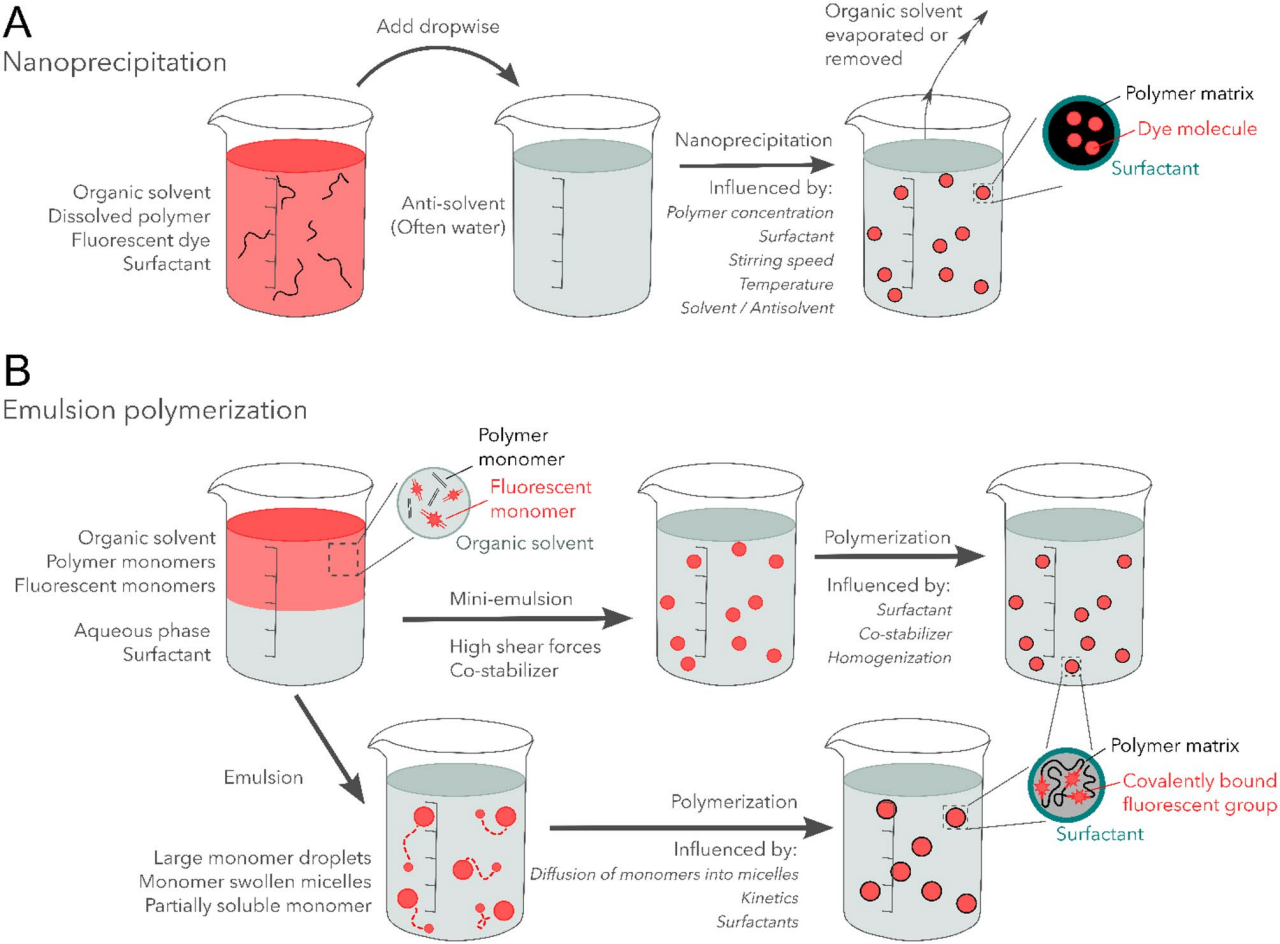
A schematic representation of synthesis techniques to obtain fluorescent NPs in the reviewed literature. **a**) Nanoprecipitation, **b**) emulsion polymerization, both conventional and mini-emulsion polymerization

During nanoprecipitation, a polymer, fluorescent dye, and sometimes stabilizer or surfactant are first dissolved in a water-miscible organic solvent, such as tetrahydrofuran (THF) or acetone. Consequently, the solution is added to water, or another polar solvent, which acts as an antisolvent because of the insolubility of the polymer in this medium. This results in supersaturation of the polymer, which leads to NPs via nucleation and growth phenomena. Figure 7a schematically illustrates these steps. To achieve high and homogeneous dye loadings, it is therefore important to have similar solubilities for the polymer and the fluorescent dye within the initial solution. It is useful to note that this process is kinetically controlled. Hence, the particle size is determined by several factors, such as the water/organic solvent ratio and polymer concentration [125, 134]. Additionally, it should be kept in mind that there is no covalent bond formation between the polymer and dye, which could result in stability issues. However, it is expected that the dyes are encapsulated within the precipitated polymer network and hence will be less prone to leaching. Extra care can be taken to use dyes that do not dissolve in water themselves, as it can be expected that once encapsulated within the (hydrophobic) polymer matrix, they won’t leach out in aqueous solutions.

Bhargava et al. encapsulated the hydrophobic fluorescent dye perylene butyl tetraester (PTEN-H) in PMMA nanoparticles via this nanoprecipitation technique, with acetone as a solvent and sodium dodecyl sulfate (SDS) as the stabilizing agent [112]. Their average particle size was 185 ± 3 nm, as determined by dynamic light scattering (DLS). Ultraviolet and photoluminescence spectroscopy were used for the determination of the photophysical properties, indicating absorption wavelengths at ~ 450 and ~ 470 nm, and emission at ~ 565 nm. These particles remained stable in deionized water for 6 months but were found to flocculate in filtered seawater due to its high ionic strength when the concentration exceeded 50 ppm. Importantly, the fluorescence intensity of the particles was high enough to allow detection with fluorescence microscopy in barnacle naupli that were exposed to the fluorescent particles [112]. 

The same method and chemicals were used by Batool et al., who incorporated PTEN-H in both PMMA and polyvinyl acetate (PVAc) [65]. Their obtained particle size as determined by DLS and SEM ranged from 60 to 135 nm for PMMA and 70–100 nm for PVA. They suggest that this size variation is caused by differences in the dye concentration. After performing coprecipitation with CaCl_2_ and Na_2_CO_3_ as a strategy to remove the NPs from water, the plastic particles ended up in the precipitate, showed significant aggregation, and remained fluorescent [65]. In another recent study, Mahadevan and Valiyaveettil used the same approach for PMMA and PVC in a toxicology study [113]. For each type of plastic, the adsorption and emission wavelengths of PTEN-H remained unchanged. Lastly, Johnson et al. employed a precipitation method to fabricate fluorescent PET NPs [114]. 

These studies demonstrate that nanoprecipitation can be used for a wide variety of plastic types. Additionally, only small amounts of dye (2–4 wt%) are used in this strategy. Surfactant-free methods might be possible candidates for synthesizing plastic nanoparticles with a closer resemblance to secondary NPs. However, the stability must be considered to prevent preliminary aggregation and dye leaching, which remains to be investigated. Moreover, surfactant-free synthesized NPs would still not have the same shape and surface groups as secondary particles. Additionally, the surfactant plays a role within the final particle size distribution, and doing surfactant-free synthesis might therefore lead to very broad particle ranges or (relatively) large particle sizes.

**Fig. 8:**
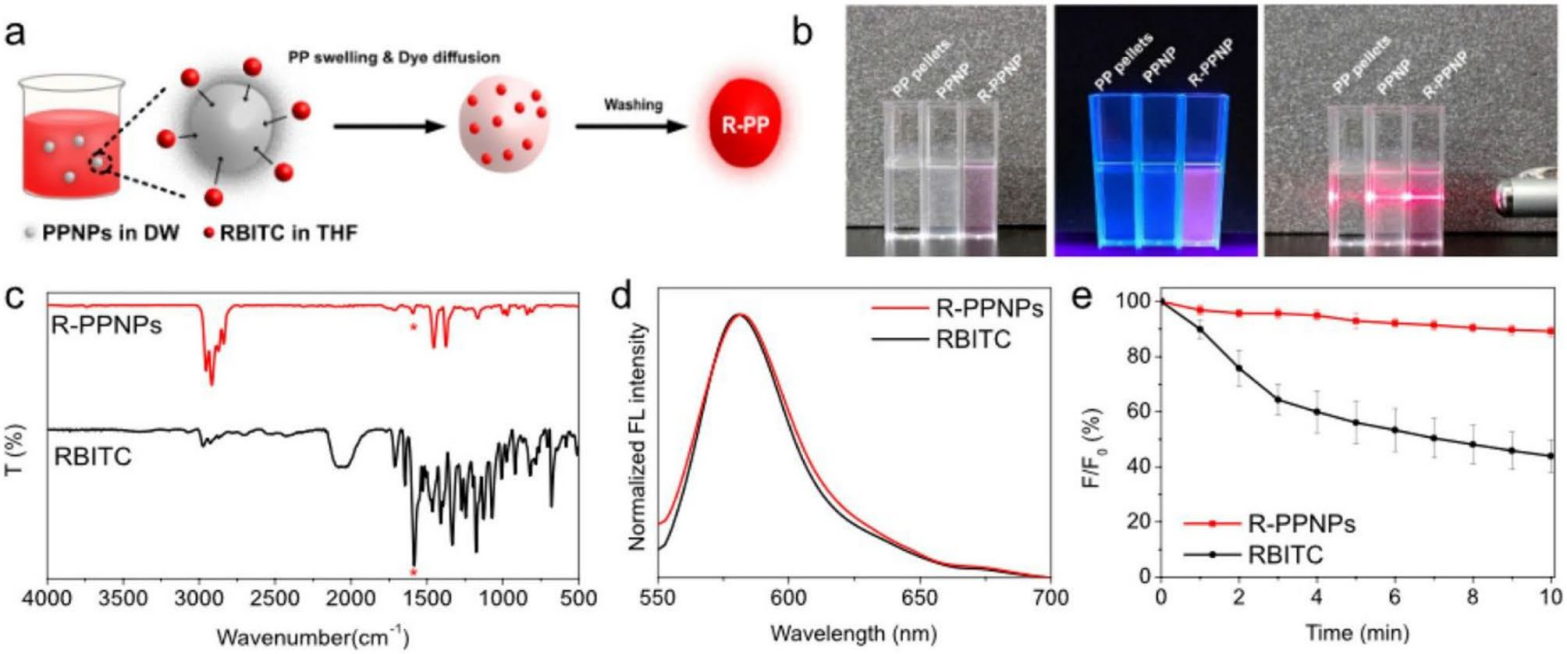
**a**) Schematic overview of the combined swelling and diffusion method done on polypropylene (PP) nanoparticles (NPs) using rhodamine B isothiocyanate. **b**) Photographs of the PP pellets, nanoparticles (NPs) and stained NPs also under UV-light and with a visible light laser beam; to demonstrate their fluorescent properties and dispersion. **c**) Fourier transform-infrared (FT-IR) spectroscopy on the stained particles shows hints of the Rhodamine-based dye, but in very low concentration as indicated by the red marker. **d**) Fluorescence spectra of both the stained particles and the pure dye. **e**) The fluorescent stability of the stained particle and pure dye. Reproduced with permission from Lee et al. [98]

Lee et al. also employed nanoprecipitation to generate PP NPs [135]. Instead of using water, they used ethanol, which is miscible with xylene, as an anti-solvent, and dissolved the PP polymer, resulting in the precipitation of the NPs. Interestingly, no fluorescent dye was included before the precipitation step. Instead, as schematically shown in Fig. 8a, a combined swelling and diffusion method was applied to “open” the particles in THF, and distilled water was used to protect the spherical shape of the particles whilst generating an environment in which the dye, rhodamine B isothiocyanate, could diffuse into the PP particles through THF. As shown in Fig. 8b, the labelled particles showed strong fluorescence and proper dispersion, whilst IR spectroscopy showed no traces of the dye within the stained plastics, highlighting the low concentration (Fig. 8c). Finally, the fluorescent stability of the NPs versus the pure dye was tested, and revealed that the particles were more stable than the pure dye.

To summarize, nanoprecipitation is a valid option to synthesize model fluorescent (nano)particles with many parameters to control outputs. Although many plastics are suitable for this strategy, it should be noted that the polyolefins, especially PE, is more challenging to use as it requires e.g. (hot) toluene or dichloromethane to dissolve that can present practical issues and hazards.

#### Conventional emulsion polymerization

Copolymerizing a fluorescent dye monomer with a plastic precursor can produce fluorescent NPs. This is significantly different from the methods before, as the covalent bond of the plastic with the dye prevents dye leaching. The only way in which the dye would be released would be through polymer degradation, which is an additional benefit as this allows for degradation studies. One approach to synthesize such particles is conventional emulsion polymerization.

As illustrated in Fig. 7b, monomers are dispersed in a surfactant-containing aqueous phase, leading to the formation of both large monomer droplets and monomer-containing micelles, as the surfactant concentration generally exceeds critical micelle concentration. After initiation, polymerization occurs in the micelles, leading to the formation of plastic nanoparticles. Monomers simultaneously diffuse from the large monomer droplets to these micelles. As the surfactant is incorporated on the surface as well, its removal is often problematic and not performed. It should be noted that emulsion polymerization can be performed without surfactants, following a different, but highly effective, (homogeneous) nucleation mechanism, in contrast to mini-emulsion polymerization (*vide infra*) that needs the surfactant for interfacial stabilization to prevent droplet coalescence and resulting rapid Ostwald ripening [128] Another challenge is the homogeneous dye loading since both conventional monomers as dye monomers have to diffuse through the aqueous phase first [125]. 

Vicenti et al. synthesized PS NPs with emulsion polymerization of styrene and the hydrophobic 7-[4-(trifluoromethyl)-coumarin] acrylamid monomer on Al_2_O_3_ nanoparticles [117]. Al_2_O_3_ NP cores are not expected to influence toxicity, but they were used for a coupled detection method. The sizes of particles with and without palmitic acid were 337 ± 9 nm and 332 ± 6 nm, respectively. Because of the low concentration of fluorescent monomers, the surface chemistry is likely not affected by the fluorophore, merely by the surfactant SDS. To further assess surface properties, an additional batch of these PS NPs functionalized with palmitic acid was used to simulate environmental degradation properties. The authors emphasize the importance of mentioning the carboxylic acid group source and use palmitic acid because of its common presence in oils and soaps [117].

#### Mini-emulsion polymerization

Another strategy for the copolymerization of fluorescent dye with regular monomers is mini-emulsion polymerization. During this operation, a surfactant-containing aqueous phase and a hydrophobic phase that contains both fluorescent dye monomers and regular monomers are subjected to mini-emulsification, resulting in submicron monomer droplets stabilized by surfactant on the surface. After initiation, polymerization converts the droplets into plastic nanoparticles, as illustrated in Fig. 7b [125]. 

One advantage of this method compared to conventional emulsion polymerization is the elimination of the diffusion of monomers through the aqueous phase, which causes inhomogeneous dye loadings in conventional emulsion polymerization. Moreover, this strategy generally results in smaller particles. However, the method is slightly more complex and expensive due to the mini-emulsification step.

This technique was used by Booth et al., who synthesized two kinds of PMMA nanoparticles with different hydrophobicity and incorporated a fluorescent monomer in both [115]. To clarify, regular PMMA was of medium hydrophobicity, and poly(methylmethacrylate-costearyl methacrylate) (PMMA-PSMA) was highly hydrophobic. The nanoparticles were synthesized via mini-emulsion polymerization and contained a small amount of the fluorescent comonomer 7-[4-(trifluoromethyl)coumarin] acrylamid, eliminating the risk of leakage. The obtained particle sizes ranged from 86 to 125 nm. Importantly, no difference was found in toxicity between the particles with and without fluorescent tags. In an earlier study, Booth et al. investigated the stability of these PMMA and PMMA-PSMA particles and a hydrophilic PMMA derivative, poly(methylmethacrylate-co-hydroxyethyl methacrylate) [115]. They found that the hydrophilic derivative was unstable in deionized water and that the stability under saline conditions depended on the surfactant for all three kinds. The particles with SDS as a stabilizing agent aggregated under these conditions, whereas the ones using Lutensol AT50 did not. It is therefore of utmost importance to choose the stabilizing agent with care, but also to realize the impact of using surfactants in general, as it completely dominates particle behavior in dispersions. Again, these stabilized particles are likely not representative of naturally occurring secondary plastics, but it is unfortunately not possible to perform this synthesis without surfactants.

Sun et al. used a mini-emulsion polymerization strategy to synthesize PS-SO_3_H and PS-NH_2_ spheres, with particle sizes of 55 ± 7 nm and 71 ± 6 nm, respectively [136]. No details about the fluorescence properties were disclosed. However, this illustrates that surface functionalization is also possible with this technique.

A slightly modified approach was performed by Cassano et al. for synthesizing fluorescent PP nanoparticles [116]. To obtain fluorescent properties, they did not use solely organic dyes, but either the organometallic dye platinum octaethylporphyrin or quantum dots. The presence of metals enabled coupled detection techniques. Additionally, they opted for the biocompatible sodium cholate as a surfactant.

### Towards realistic secondary plastic nanoparticles

Three studies have explored strategies to produce fluorescent NPs with a closer resemblance to secondary NPs. First, Caldwell et al. developed heterogenous and surfactant-free PP, PET, and PS NPs via melt-processing and milling, with the fluorescent 1,4-bis(-cyano-4-methoxy-styryl)-2,5-dimethoxybenzene dye [137]. The results confirmed that the particles do not show dye leakage in a hydrophilic cell cultivation medium. However, the brute forces during milling might not properly reflect environmental forces, so the resemblance of the resulting morphology and structure of these particles to secondary NPs should be assessed. Second, Annenkov et al. produced PVC, PS, and PMMA with the water-insoluble, fluorescent dibenzylfluorescein dye [36]. Consequently, the fluorescent macroplastics were subjected to mechanical forces that simulate environmental conditions to render fluorescent NPs. No leakage of the fluorescent dye was observed in water. Both frameworks, however, disregard the potential stability issues in hydrophobic parts of cells. In addition, the surface groups of these particles are not likely to show resemblance to secondary NPs. However, all in all, these particles approach secondary NPs to a greater extent than the aforementioned methods, where only spherical-like particles were considered.

A very promising synthesis method is demonstrated by Muff et al., who use confined impinging jet mixing to produce PS, PE, PP and PET NPs with sizes below 100 nm. Several fluorophores could be implemented in their particles, and to bridge the gap towards secondary NPs they changed their shape in a post fabrication process [138]. This is a promising method to turn primary NPs in more realistic model systems, while still maintaining the high amount of control over the initial particles. If this would be combined with e.g., UV-weathering, the particles might approach secondary NPs. However, it is hard to verify if all these studies indeed closely resemble secondary NPs, as the number of spectro-microscopic studies on environmental NPs is limited. Nevertheless, the efforts to approach secondary NPs for a variety of polymers are vital.

### Knowledge gaps

**Fig. 9:**
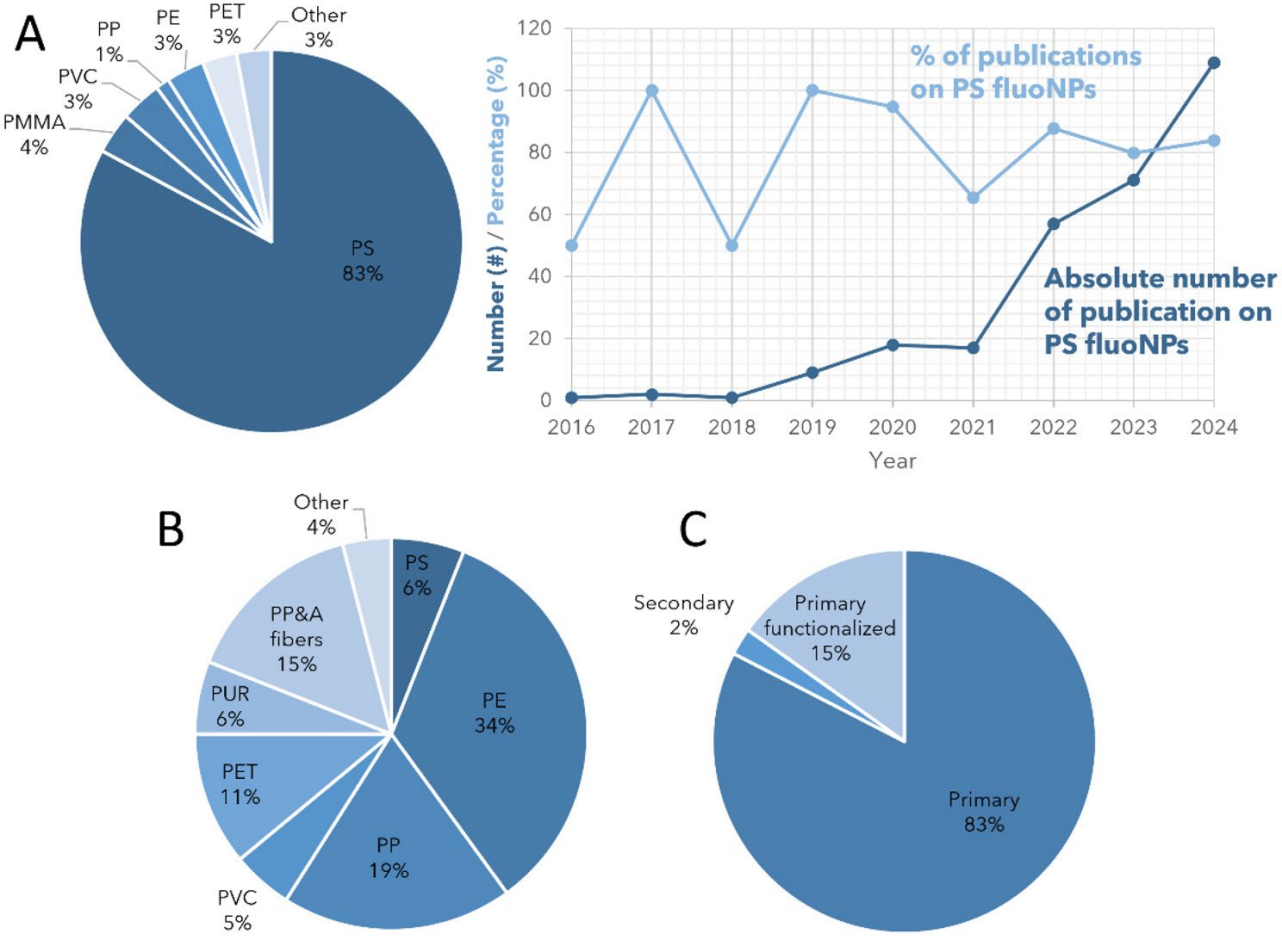
Distribution of plastic types, **A**) encountered for NPs in reviewed literature in total and for polystyrene (PS) specifically over the years. **B**) The distribution of plastic waste in 2015, which was prior to our selection of literature [1]. **C**) The types of NPs found in the reviewed literature

From the current literature assessment, we see that a large gap exists between the plastic particles investigated in exposure studies and the plastic particles expected in the environment. As highlighted before, the current literature is heavily biased towards PS NPs. Figure 9a shows the difference between the distribution of plastic types in the reviewed literature and the plastic waste distribution published in 2015 (Fig. 9b), which we specifically picked here as this was at the starting point of our assessed literature. In this current selection of literature for this work, PS was used in 83% of the studies. The real plastic waste distribution, which is estimated to be 36% PE, 21% PP, 12% PVC, and less than 10% for PET, PS, and PUR each [1]. Even if this plastic waste distribution would not fully simulate the ratio of polymers we are exposed to on a daily basis due to single-use plastics, water and air pollution, etc., this gap is still essential, since for MPs, it is already known that the polymer type influences the specific toxicity [11]. The plot in Fig. 9a shows the total number of publications from the assessed literature using fluorescent PS spheres (dark blue) and the ratio of all reported fluorescent NPs being PS (light blue). Interestingly, although the total amount of publications has risen, and it is often discussed that PS should not exclusively dominate the research field, the total number of publications on PS has increased in a similar trend over recent years showing that the percentage of PS (especially since 2019, where there were more than 10 publications per year) is rather constant. This can be easily explained by the accessibility of the PS spheres from commercial sources, and although moving towards new model particles would provide valuable information, screening and initial tests are often performed on the PS NPs.

The largest gap remains the discrepancy between primary and secondary NPs. In Fig. 9c the distribution between primary and secondary particles is shown. In 98% of the reviewed studies primary fluorescent particles were used. Clearly, the actual synthesis of realistic secondary fluorescent particles remains a challenge. Furthermore, secondary plastic particles are not often reported as most of the detection techniques applied for environmental samples are still leaning towards mass-based analysis instead of micro-spectroscopy, and fluorescence specifically [21]. 

The importance of surface groups and the consequent charge has already been established in prior research [90, 91, 136]. As shown in Fig. 9c and 15% of the reviewed papers used functionalized plastic nanoparticles to investigate these effects. For example, Sun et al. demonstrated that positively charged PS-NH_2_ accumulates on a different location in plants than negatively charged PS-SO_3_H [136]. Also in human HepG2 cells, it was found that surface functionalization with -COOH or -NH_2_ groups increased NP accumulation and decreased cell viability [90]. In a study on krill, Bergami et al. observed aggregation of the anionic carboxylic PS NPs in seawater, while the cationic amino-PS NPs remained their original size, showing their different behavior [91]. Some limitations of these kinds of studies are the lack of similarity of these surface groups to the groups that are introduced by degradation and the fact that these particles are still spherical and stabilized by ligands; but on the other hand they demonstrate the importance of chemical functionalization on the surface of particles even if the morphology remains constant, proving that surface groups should be considered as one of the major parameters to study in the NP research field.

In a review article by Zhang et al., the degradation mechanisms in light and heat of PS, PP, PET, and PE are presented [9]. It is apparent that most pathways lead to C = O or C-OH groups. It should further be realized that degradation caused by UV leads to radical mechanisms and that the mechanism and surface groups depend on the type of plastic and conditions. Generally, degradation is classified into two classes. Abiotic degradation concerns UV radiation, mechanical stress (rocks, waves), heat, and chemicals. Biotic degradation might concern biodeterioration, bio-fragmentation, and assimilation. When simulating environmental degradation, all these different factors should therefore be considered. Not just mechanical stress, as was done by Annenkov et al. in their efforts to estimate the number of sub-microplastics in the environment by degrading fluorescent macroplastic, although it is a very good start [36]. Moreover, the spherical shape of primary nanoparticles is inherently different from fragmented secondary nanoparticles. This might be a considerable factor in toxicology, as was highlighted in a recent review by Kögel et al. [11] The reader is referred to this article for a greater understanding of factors that determine the toxicity of MPs and NPs. However, particle shape has not been considered yet in the current dataset of fluorescent nanoparticles.

Lastly, it is expected that NPs can adsorb other pollutants as well. This has been examined by Trevisan et al., as they studied the adsorption of a fluorescent class of pollutants, polycyclic aromatic hydrocarbons (PAH), on NPs [139]. Compared to non-adsorbed PAH, they found that adsorbed PAH accumulated in different areas in zebrafish. Interestingly, the nanoparticles might also adsorb molecules that decrease the toxicity of the NPs. For example, Fadare et al. illustrated how adsorption of humic acid decreased the toxicity of NH_2_-PS NPs in zebrafish [99]. However, these effects might again depend on the surface groups and the resulting charge. Therefore, it is of utmost importance to find ways to study the toxicology and behavior of secondary NPs.

### Recommendations – fluorescent nanoplastics

1. Move away from commercial PS beads for risk assessment studies.
2. (Mini-)emulsion polymerization seems the most promising technique to generate NPs without dye leakage. However, the polymer properties should be tested when introducing a different monomer within a polymer matrix, and the method is limited to certain polymers.
3. Nano-precipitation offers large control over particle properties and is widely applicable to different polymers, dyes and markers.
4. Test stability of fluorescent NPs under several, but most important, relevant conditions.
5. Optimize methods to produce fluorescent secondary NPs.

## Concluding remarks, recommendations and outlook

### Concluding remarks

Fluorescence-based analytical methods provide potential for the detection of NPs in environmental samples and organisms, but more importantly, a well-established methodology for exposure assays using model fluorescent particles. Fluorescent NPs can be obtained in various ways. Commercial polystyrene (PS) beads are most commonly used, but they might be prone to leakage, that is unfavorable in toxicology studies. However, this depends on the synthesis route, that is often not disclosed by the manufacturer. In addition, the large quantity of research on PS is not representative of real-life scenarios at all. Synthesizing NPs via nanoprecipitation already allows for the use of a wider variety of plastic types, although the particles might still suffer from dye instability drawbacks. These drawbacks are not encountered for emulsion polymerization, as a covalent bond is formed between the fluorophore and plastic, but unfortunately the selection of polymers and dyes is more limited for this approach. Lastly, lipophilic staining can be employed to detect either environmental NPs or non-fluorescent particles that were tested in model studies. Nevertheless, these methodologies are mostly established for MPs, and not for NPs that are intrinsically more challenging to detect with fluorescent microscopy techniques due to their smaller sizes.

Most apparent is the heavy bias towards using primary NPs, as opposed to using secondary NPs. Of the afore-mentioned methods, only lipophilic staining might be suitable for secondary NPs. Nevertheless, the difference in the shape, surface groups, and stability of primary versus secondary NPs likely result in completely different particle behavior and toxicity. Therefore, it is of high interest to design more secondary-like particles that still have trustworthy fluorescent properties.

### Recommendations for current and future research

**Fig. 10:**
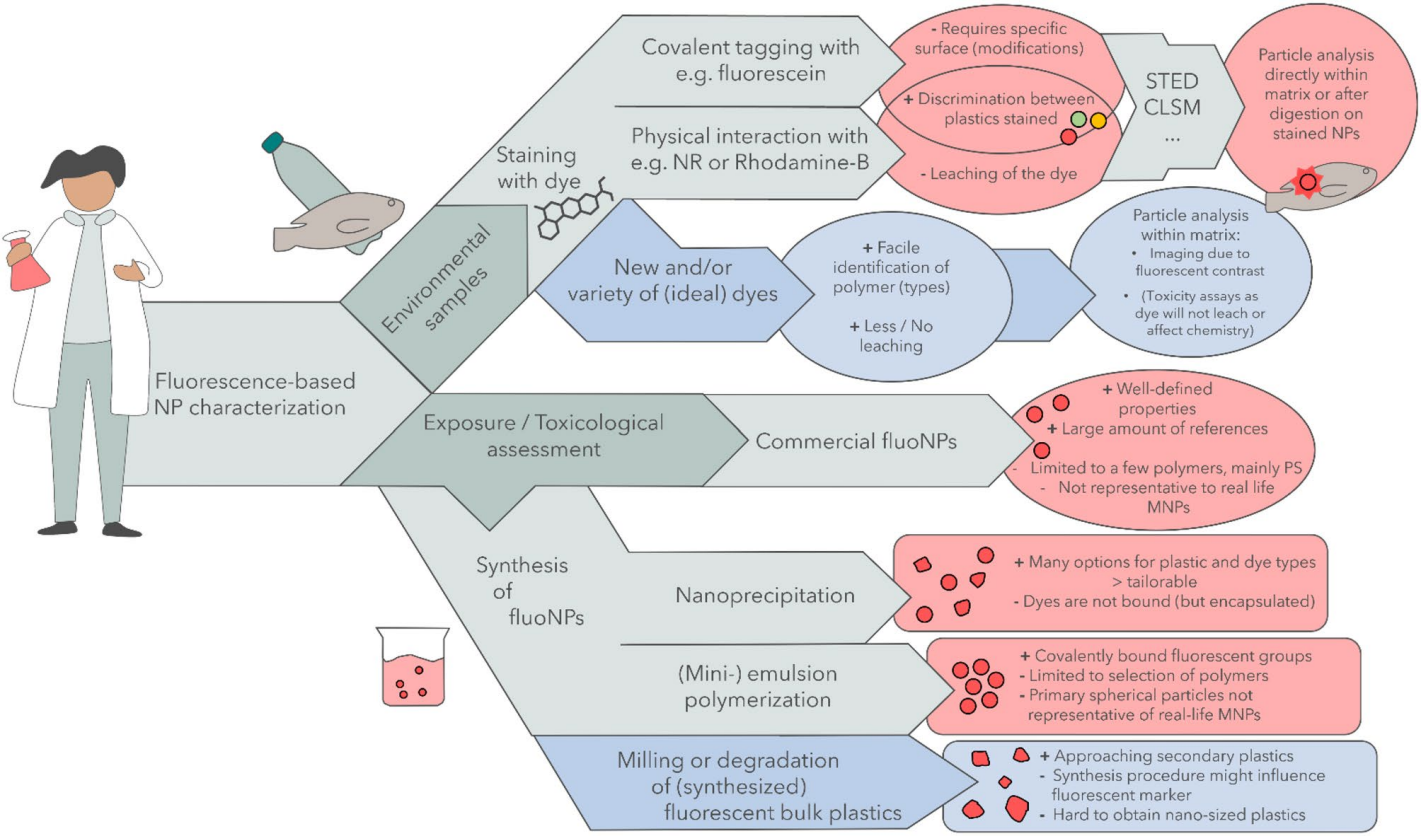
Suggested flow chart for researchers wanting to use fluorescence-based analytical techniques and particles within nanoplastic (NP) research. The red circles explain positive and negative sides of choosing a certain route, whereas light-blue indicates that this route is not (commonly) established yet within the assessed literature

It is critical to expand on the currently used synthesis and labelling methods and focus on producing a diverse range of fluorescent (secondary) NPs and suitable dyes. For this, it is important to keep the purpose of the fluorescent NPs in mind. In this review article, we present a plausible but effective “roadmap”, considering the two main applications: detection of secondary NPs in environmental samples, and the use and detection of NPs in exposure studies. This flow chart is shown in Fig. 10. The green routes follow existing pathways, whereas the blue routes offer alternative or ‘ideal’ scenarios. The red boxes state the current outcomes of the routes, with pros and cons.

For detection of secondary NPs in the environment, a lipophilic staining procedure is recommended at this stage. Currently, NR is used as the main staining dye for NPs, but a study dedicated to selecting different dyes would be beneficial, as some dyes might selectively stain one plastic-type. It is crucial to use secondary NPs and different plastic types in this study, as the surface properties might play a significant role in fluorescence intensity. Based on the current research on MPs, it would be highly interesting to study heat treatment and fluorescence lifetime. This would enable determining the selectivity of a dye towards a specific plastic-type and allow the discrimination of different types of plastic in real-life environmental samples.

It should also be stressed that most current staining/dyeing methods are optimized for spiking laboratory samples, not work with environmental samples (sediment, wastewater, biota) and this limits their applicability and ecological relevance. Therefore, besides the plastic/dye combination studies described above, it is recommended that the field moves towards methods that selectively stain MNPs in complex mixtures without high false-positive rates. Hence, the dyes or probes used must be stable under natural environmental conditions (sunlight, salinity, microbial activity). Ideally, these approaches also work in vivo for uptake studies without having considerable toxic effects. This could link in vivo particle tracking to meaningful biological/ecological outcomes and not just particle detection.

Covalent tagging is a specific, promising alternative to lipophilic/physical staining methods. However, understanding surface chemistry and properly linking the right dye to any plastic is a challenge and there is a large benefit of having covalently bound fluorescent molecules. Still, this methodology also requires careful leaching tests as the bonds are mostly on the surface; exactly where the particles will also interact with the medium to which they are exposed. Finally, an important aspect in the comparison of dyes would be to use a quantitative technique, such as spectro-fluorometry. As sketched in the blue route in Fig. 10, staining could theoretically remove the need to synthesize bottom particles all together. If the staining dye would not leach, secondary particles could be generated and dyed, that would produce the most realistic model particles for toxicological and exposure assays.

In consideration of exposure studies, other factors should be contemplated, mainly stability. The most optimal solution to dye leaching from the synthesized particles is to form covalent bonds between fluorophore and plastic. Co-polymerization strategies would be most favorable, as the surface groups of the particles would remain intact as well. However, in the presented selection of strategies, only primary NPs can be formed. That is why a completely new method is provided here. In this hypothetical method, first, a fluorescent macroplastic is formed via a general polymerization technique, but with the addition of a copolymerizing fluorescent monomer. Consequently, secondary NPs are created by exposure of this plastic in water to realistic environmental conditions, like weathering conditions and mechanical stress [9, 140]. Lastly, organisms are exposed to the resulting aquatic solution that contains fluorescent NPs. One critique of this strategy could be that it does not provide fundamental insight into the effects of particle size, as is often done in toxicology studies. However, the heterogeneity of NPs is also encountered in environmental samples. So far this had not been considered in toxicology studies, while it might be contributing to their behavior and toxicity [7]. Additionally, (cascade) filters can be utilized to select NPs within specific size ranges.

Nevertheless, nanoprecipitation should not be overlooked, as it allows for more variety in the resulting fluoNPs, and this is (especially with the established field mainly focusing on PS) very important for research within the near future. The high level of control over particle parameters also enables the generation of a particle-toolset that could reveal mechanistic insights within organism-particle interactions. The flexibility and rather easy synthesis procedure makes this method more than suitable for studying marked particles other than PS. It is expected that both approaches will contribute to insight that allows a better understanding of the gap between NPs and MPs on the one hand and primary and secondary particles on the other hand. For both methods, progression towards more (ecologically) relevant model particles is possible, as post-synthetic weathering simulations, or chemical modification through e.g. surface oxidation, would already provide a departure from the pristine particles that are very unlikely to be found in the environment.

### Outlook

In recent years, great advances have been made in terms of exposure studies using fluorescent NPs and the synthesis of fluorescent NPs. As the research on such particles is exponentially increasing, as shown in Fig. 1, it is unavoidable that the coming years will reveal even more insights into these topics. It will be essential, and also challenging, to harmonize the most optimal methods to stain, synthesize and detect fluorescent NPs. Only then, will the field of (fluorescent) NPs develop towards a broad, yet detailed understanding of the potential effects of NPs within experimental inconsistencies, enabling direct comparison and complementarity of work from diverse sources. In general, fluorescence detection methods have shown to be a fantastic asset within the field of NP exposure studies, and it is likely that this will not change. Beyond organic fluorophores, there is active work on metal- or rare-earth–tagged model NPs (e.g., Au-core or quantum-dot/metal-doped polymers) because they enable ultrasensitive mass-based readout (ICP-MS) and EM co-localization; however, these tags can change particle density and surface chemistry, carry tag-specific toxicity, and decouple the readout from the plastic itself (metals are quantified, but they can detach), thereby confounding dose–response interpretation. In contrast, fluorescent labeling, ideally with covalently integrated dyes, supports minimal perturbation, and enables live-cell compatible imaging and multiplexing while avoiding metal background and heavy-atom quenching effects. With rigorous QA/QC (leaching, photostability, matrix controls), fluorescence therefore offers the more suitable primary strategy for isolating polymer-driven biological effects, while metal/rare-earth tags are best reserved for orthogonal validation and mass-balance checks. The accessibility and relative ease in which the particles can be distinguished from their surrounding matrices foreshadows that these methodologies will play a vital role in understanding the effects of NPs for many decades to come.

## Data Availability

The datasets used and/or analysed during the current study are available from the corresponding author on reasonable request.

## Abbreviations

AFM: Atomic force microscopy
CA: Cellulose acetate
CLSM: Confocal laser scanning microscopy
CUSP: European research cluster to understand the health impacts of micro- and nanoplastics
DLS: Dynamic light scattering
EM: Electron microscopy
EPS: Expanded polystyrene
HDPE: High density polyethylene
LDPE: Low density polyethylene
MNPs: Micro and nanoplastics
MPs: Microplastics
NPs: Nanoplastics
NR: Nile red
PAH: Polycyclic aromatic hydrocarbons
PAN: Polyacrylonitrile
PET: Polyethylene terephthalate
PiFM: Photo-induced force microscopy
PMMA: Polymethylmethacrylate
PMMA-PSMA: Poly(methylmethacrylate-costearyl methacrylate)
PP: Polypropylene
PS: Polystyrene
PTEN-H: Perylene butyl tetraester
PTFE: Polytetrafluoroethylene
PUR: Polyurethane
PVAc: Polyvinylacetate
PVC: Polyvinylchloride
QA/QC: Quality assurance / quality control
RESOLFT: Reversible saturable optical linear fluorescence transitions
SDS: Sodium dodecyl sulfate
SEM: Scanning electron microscopy
SMLM: Single-molecule localization microscopy
SPT: Single particle tracking
STED Microscopy: stimulated emission depletion microscopy
TEM: Transmission electron microscopy
THF: Tetrahydrofuran
